# The relationship between nut consumption and premature coronary artery disease in a representative sample of Iranians: Iran-premature coronary artery disease (IPAD) study

**DOI:** 10.1017/S1368980023002392

**Published:** 2023-12

**Authors:** Noushin Mohammadifard, Ghazaal Alavi Tabatabaei, Fahimeh Haghighatdoost, Ehsan Zarepur, Fatemeh Nouri, Sahel Javanbakht, Fereidoon Nouhi, Hassan Alikhasi, Tooba Kazemi, Nahid Azdaki, Nahid Salehi, Kamal Solati, Masoud Lotfizadeh, Samad Ghaffari, Elmira Javanmardi, Arsalan Salari, Mostafa Dehghani, Mostafa Cheraghi, Ahmadreza Assareh, Habib Haybar, Seyedeh Mahdieh Namayandeh, Reza Madadi, Nizal Sarrafzadegan

**Affiliations:** 1 Interventional Cardiology Research Center, Cardiovascular Research Institute, Isfahan University of Medical Sciences, Isfahan, Iran; 2 Isfahan Cardiovascular Research Center, Cardiovascular Research Institute, Isfahan University of Medical Sciences, Isfahan, Iran; 3 Department of Cardiology, Medicine School, Isfahan University of Medical Sciences, Isfahan, Iran; 4 Rajaie Cardiovascular Medical and Research Center, Iran University of Medical Sciences, Tehran, Iran & President of Iranian Network of Cardiovascular Research (INCVR); 5 Heart Failure Research Center, Isfahan Cardiovascular Research Institute, Isfahan University of Medical Sciences, Isfahan, Iran; 6 Iranian Network of Cardiovascular Research; 7 Cardiovascular Diseases Research Center, Birjand University of Medical Sciences, Birjand, Iran; 8 Clinical Research Development Unit, Imam Reza Hospital, Birjand University of Medical Sciences, Birjand, Iran; 9 Cardiovascular Research Center, Health Institute, Kermanshah University of Medical Sciences, Kermanshah, Iran; 10 Department of Psychiatry, Shahrekord University of Medical Sciences, Shahrekord, Iran; 11 Social Determinants of Health Research Center, Shahrekord University of Medical Sciences, Iran; 12 Cardiovascular Research Center, Tabriz University of Medical Sciences, Tabriz, Iran & Member of the INCVR; 13 Department of Cardiovascular Medicine, Heart Center, Maragheh University of Medical Sciences, Amiralmomenin Hospital, Iran; 14 Department of Cardiology, Healthy Heart Research Center, Heshmat Hospital, School of Medicine, Guilan University of Medical Sciences, Rasht, Iran; 15 Department of Cardiovascular Research Center, Shahid Rahimi Hospital, Lorestan University of Medical Science, Khorramabad, Iran; 16 Atherosclerosis Research Center, Ahvaz Jundishapur University of Medical Sciences, Ahvaz, Iran & Member of the INCVR; 17 Yazd Cardiovascular Research Center, Shahid Sadoughi University of Medical Science, Yazd, Iran; 18 Department of Cardiology, School of Medicine, Zanjan University of Medical Sciences, Zanjan, Iran; 19 Faculty of Medicine, School of Population and Public Health, University of British Columbia, Vancouver, Canada

**Keywords:** Premature coronary artery disease, Nuts, Ethnic group, Iran

## Abstract

**Objective:**

The cardioprotective effects of nuts are well established. However, the positive impacts of nuts in preventing CVD at a younger age, a condition known as premature coronary artery disease (PCAD), is still debated. Therefore, we aim to determine the association between nuts and PCAD occurrence and its severity in different Iranian ethnicities.

**Design:**

This case–control study was conducted within the framework of the Iran-premature coronary artery disease (I-PAD) study, an ongoing multi-centric study on Iranian patients of different ethnicities.

**Setting:**

This multi-centric case–control study was conducted in among 3253 persons under the age of 70 years in women and 60 years in men from different ethnicities in Iran.

**Participants:**

Information on nut consumption was collected using a validated FFQ. Subjects were selected from among the candidates for angiography. Cases were those whose coronary angiography showed stenosis of more than 75 % in at least one vessel or more than 50 % of the left main artery, while the control group participants had normal angiography results.

**Results:**

In the crude model, compared to the first quartile, the highest quartile of nut consumption was significantly associated with a lower risk of PCAD (OR = 0·26, 95 % CI (0·21, 0·32); *P*_for trend_ = 0·001). In the top quartile of nut intake, a substantial decrease in PCAD was observed after controlling for putative confounders (OR = 0·32; 95 % CI (0·24, 0·43); *P*_for trend_ = 0·001). Additionally, a 75 % decrease in the risk of severe PCAD was observed in the participants in the highest quartile of nut intake.

**Conclusion:**

A significant inverse association was observed between nut intake and the risk and severity of PCAD in the Iranian population. Large-scale clinical trials are required to confirm these findings.

Coronary artery disease (CAD) is the most prevalent and widespread heart disease, which is responsible for the majority of deaths globally and causes over 350 000 fatalities annually^(1)^. It is characterised by occlusion of the coronary arteries and results in a demand–supply mismatch of oxygen^(2)^. Premature coronary artery disease (PCAD), which occurs in younger ages, is the main cause of loss of potentially productive years of life and imposes an enormous economic burden on healthcare systems^(3)^. Although the mortality rate of CAD has dropped dramatically in recent years, there has been no comparable decrease in PCAD mortality among patients^(1)^. Therefore, the detrimental effects of PCAD can be mitigated by altering modifiable risk factors^(3,4)^. Most of preventive measures are based on lifestyle changes, particularly eating habits^(2)^.

Previous research has shown that the dietary patterns such as Mediterranean and Dietary Approaches to Stop Hypertension (DASH) high in nuts and seeds can reduce the risk of cardiovascular disease (CVD), stroke^(5)^ and CAD^(6)^. The cardioprotective effects of nuts and seeds are attributable to their high unsaturated fat content, mainly in the form of mono unsaturated fatty acids (MUFA), primarily oleic acid and poly unsaturated fatty acids (PUFA), as well as proteins, antioxidants, vitamins, and minerals such as magnesium and potassium^(7)^. These studies indicated that a higher daily consumption of nuts was associated with a lower CVD risk factors at a young age (< 30 years old)^(8)^ and older adults (> 35 years old)^(6,7,9–11)^. Additionally, favourable effects of nuts on lipoprotein, TAG and total cholesterol (TC) have been propounded individuals, who added nuts into their daily diet^(11,12)^. This positive impact was also noticed when studying younger individuals aged between 16 and 25 years^(13)^.

Due to the deleterious effects of CVD on the healthcare system, strategic initiatives to limit its incidence, particularly in the younger section of society, are imperative^(14)^. Although CVD in young adults are increasing, the frequency of people under 60 years of age being affected by CAD is frequently underestimated, most likely because these patients are mainly asymptomatic^(15)^. According to previous studies, the primary pathophysiology of CAD is the same in young and elderly patients (younger or older than 45 years in males and 55 years in females)^(16)^. However, risk factors, clinical presentation and angiographic pattern may differ significantly^(16)^. Therefore, more clinical research is needed to target PCAD and explore whether dietary modification plays a role in reducing its incidence. Additionally, the diversity of socio-economic background, dietary preferences^(17)^, risk factors and cultural habits among Iranian ethnic groups warrants research taking into account these differences. Therefore, in this study, we evaluated whether a higher consumption of nuts is associated with the risk and severity of PCAD.

## Methods

### Study population

This case–control study was conducted within the framework of the Iran premature coronary artery disease (IPAD), an ongoing multi-centric study on Iranian patients of different ethnicities. Details regarding the methodology of this study have been described previously^(17)^. Briefly, patients were selected from fifteen cities based on race distribution (Fars, Azari, Arab, Lor, Gilak, Balouch, Turkaman, Qashqai and Bakhtiari). Our inclusion criteria were (1) having coronary angiography; (2) being under the age of 70 years for women and 60 years for men, (3) being a member of one of the ethnic groups of interest and (4) being aware of their parental ethnicity. Cases were defined as having an occlusion of at least one coronary artery equal to or greater than 75 %, or a left main coronary artery of equal to or greater than 50 %. Normal coronary arteries were considered as healthy and control groups^(17)^. Patients were excluded from the study if they had previous history of documented CAD, including coronary artery bypass surgery, balloon angioplasty or percutaneous coronary intervention. In the present study, a total of 3253 participants were included in this study. All patients provided written informed consent for this study, which was approved by the Ethics Committee of the Isfahan University of Medical Sciences (IR.MUI.REC.1396.2.055).

### Data collection

The main cardiac catheterisation centres in each city with a dominant ethnic group were chosen and asked to recruit individuals who met the inclusion criteria. After explaining the details of the study to the participants and obtaining their consent, they were enrolled in the study. Information regarding demographic variables, such as age, sex, ethnicity, religion, education, income and marital status, was gathered by trained interviewers and recorded. Lifestyle factors, including smoking habits, alcohol consumption, drug consumption and physical activity, were assessed using validated questionnaires. Trained personnel followed the normal protocols for measuring height and weight, waist circumference (WC) and the BMI was calculated by dividing weight (kg) by the square of height (m^2^). A 12-h fasting blood sample was collected to measure triacylglycerol (TAG), TC, HDL-cholesterol, LDL-cholesterol and fasting blood sugar (FBS). Diabetes mellitus (DM) was defined as having FBS ≥ 126 mg/dL or consuming any antidiabetc agent, Hypertension (HTN) was defined as having systolic blood pressure (SBP) ≥ 130 mmHg or diastolic blood pressure (DBP) ≥ 80 mmHg or consuming any anti-hypertensive agent. Hypercholesterolemia was defined as having TC ≥ 200 mg/dL or consuming any cholesterol lowering agents.

### Dietary assessment

A validated 110-item semi-quantitative food frequency questionnaire (FFQ) was used to evaluate participants’ regular dietary intake during the previous year^(18)^. Each product was analysed based on a common serving size, and participants were given nine options for stating how frequently they typically consume each food item, ranging from never/seldom to more than six times per d. The average intake of each food item (g/d) for each participant was calculated based on the weight of each serving and the frequency of consumption. Then, using Nutritionist IV software modified for Iranian cuisine, energy and nutritional intake were determined.

### Statistical analysis

Mean and standard deviations and percentages were used to summarise the continuous and categorical variables, respectively. One-way ANOVA was used to compare the mean quantitative variables between the quartiles of nut intake. When the data did not meet the parametric test assumptions, the Kruskal–Wallis test was used. Qualitative variables for different groups were compared using the chi-square (or Fisher’s exact if required) test.

Simple or multiple univariate logistic regression with a logit link function was performed to examine the association between PCAD and nut intake quartiles. In addition, to evaluate the relationship between PCAD severity and nut intake quartiles, simple or multiple univariates ordered logistic regressions was used. Crude and adjusted odds ratio (OR) and 95 % confidence interval (CI) are presented. The lowest quartile of nut intake was used as the reference. In the first model, the relationship between nut intake and the PCAD was estimated without adjustment. In the second model, we assessed this association by adjusting for sex and age. Further adjustments were based on education, smoking (never/ex-smoker/current smoker) and physical activity (METS/min/week). Additional adjustments were made for energy (kcal/d) and carbohydrate (% of energy). In the final model, BMI (kg/m^2^), hypertension (yes/ no), diabetes mellitus (yes/ no), hypercholesterolemia (yes/ no) and aspirin use (yes/no) were adjusted. Every statistical analysis was done on the base of 5 % error via SPSS for Windows version 23 (SPSS Inc.).

## Results

A total of 3253 subjects were included in this study of which 1163 were in the control group and the remaining 2090 were in the case group. Of the control and study groups, 36·3 % and 65·2 %, respectively, were men. The mean and standard deviation age in the control group was 52 ± 8·4 years, while for those in PCAD group was 55 ± 7·2 years.

Table 1 shows the general characteristics of the individuals in the PCAD and control groups by quartile of nut intake. In both groups, participants in the highest quartile of nut intake had higher alcohol consumption (*P* < 0·05) and were more likely to be male but less likely to use any kind of diabetic medication (*P* < 0·05). In the PCAD group, participants in the top quartile had lower TAG levels (*P* = 0·036) and were less likely to use antihypertensive (*P* = 0·017) and lipid-lowering medications (*P* = 0·023). The ethnicity distribution varied across quartiles in the PCAD group (*P* = 0·002). No differences were observed in terms of SBP, DBP, BMI, WC, TC, LDL-cholesterol and HDL-cholesterol across the quartiles of nut in any of the groups. Nevertheless, SBP and DBP were lowest in the second and first nut intake quartiles, respectively. Patients with PCAD and higher nut consumption had lower TC and LDL-cholesterol levels.

**Table 1:**
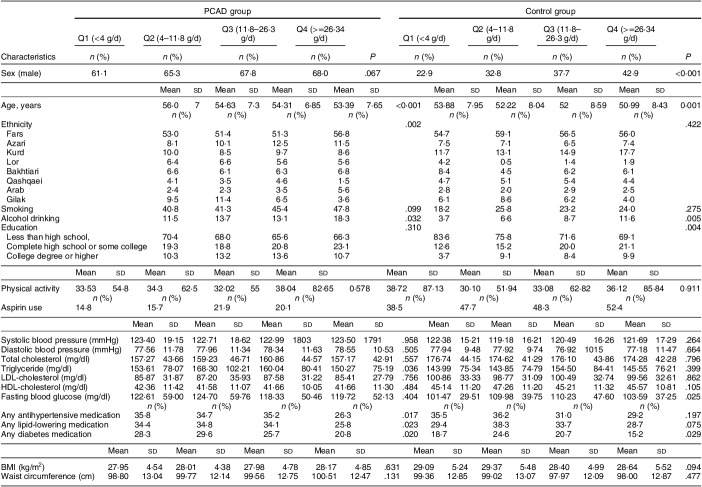
General and demographic variables in subjects with and without premature coronary artery disease based on quartiles of nuts and seeds consumption

The dietary intakes of participants in the cases and control groups are illustrated in Table 2. In both groups, higher nut intake was significantly associated with higher carbohydrate, MUFA, PUFA, total fat, total energy, red meat and sweets intake (*P* < 0·001).

**Table 2:** Dietary intake in subjects with and without premature coronary artery disease based on quartiles of nuts and seeds consumption

Nutrients and food groups	PCAD group									Control group								
	Q1 (<4 g/d)		Q2 (4–11·8 g/d)		Q3 (11·8–26·3 g/d)		Q4 (>=26·34 g/d)		P*	Q1 (<4 g/d)		Q2 (4–11·8 g/d)		Q3 (11·8–26·3 g/d)		Q4 (>=26·34 g/d)		P*
	Mean	SD	Mean	SD	Mean	SD	Mean	SD		Mean	SD	Mean	SD	Mean	SD	Mean	SD	
Energy (kcal/d)	1848·9	795·66	2003·87	729·25	2156·42	804·66	2386·11	880·59	<0·001	1730	727·25	1801·4	558·75	2000·9	675·5	2375	856	<0·001
Carbohydrate (% of energy)	53·63	9·14	52·9	8·52	51·15	8·27	46·55	7·84	<0·001	51·13	15·86	50·41	8·81	49·24	7·78	46·12	8·01	<0·001
Protein (% of energy)	16·27	3·56	15·98	3·11	16·11	2·86	15·86	2·94	0·31	16·35	3·41	16·58	3·22	16·43	2·97	16·18	2·57	0·42
Total fat	34·44	9·7	35·59	8·92	37·05	8·86	41·69	8·22	<0·001	37·56	9·57	37·5	8·84	38·88	8·38	41·84	8·23	<0·001
SFA (% of energy)	14·61	5·62	14·45	5·09	14·1	5·02	13·66	4·16	0·167	14·61	5·17	14·07	4·75	13·49	4·39	13·09	4·23	0·001
MUFA (% of energy)	10·49	3·73	10·76	3·42	11·15	3·44	12·68	3·27	<0·001	11·01	3·5	10·8	2·82	11·23	2·94	12·67	3·21	<0·001
PUFA (% of energy)	7·61	3·16	8·65	2·83	9·89	3·14	12·76	3·68	<0·001	9·55	3·66	9·85	3·53	11·46	3·4	13·51	3·85	<0·001
Fibre (g/d)	19·23	9·42	20·59	8·73	22·36	9·6	24·13	10·64	0·831	19·01	9·92	19·46	7·98	21·52	9·1	24·90	9·79	<0·001
Food group (g/d)																		
Vegetables	237·14	147·56	230·4	130·11	278·36	162·49	325·13	155·85	0·831	358·78	177·5	344·58	158·01	352·13	155·28	360·3	163·1	0·831
Fruit	166·3	102·81	175·17	91·77	207·31	111·19	243·19	130·24	<0·001	253·98	123·23	258·47	98·14	276·46	103·05	285·42	117·33	0·01
Legumes	40·69	39·31	44·58	45·78	55·7	51·48	66·98	64·69	<0·001	75·01	86·41	71·48	46·99	77·59	51·13	77·6	64·58	0·213
Whole grains	105·94	127·56	107·76	117·47	123·25	135·68	114·64	130·42	0·060	113·79	132·73	115·4	147·75	131·64	146·03	145·71	148·33	0·001
Refined grains	223·57	151·38	257·28	179·27	240·01	167·21	245·45	162·7	0·009	211·66	152·98	212·57	126·49	225·99	150·28	246·67	179·78	0·071
Non-hydrogenated vegetable oils	15·34	15·14	16·61	12·87	18·24	16·69	21·48	18·9	<0·001	23·57	19·3	22·69	16·46	23·49	14·57	22·06	14·98	0·410
Hydrogenated vegetable oils	5·5	10·9	5·59	11·64	5·23	11·41	5·21	9·02	0·526	3·25	6·63	2·78	4·12	2·29	4·03	2·77	4·65	0·398
High-fat dairy products	76·57	107·59	90·86	112·45	88·12	111·49	95·70	126·23	0·008	63·23	106·44	66·28	97·5	63	87·74	77·34	104·88	0·024
Red meat	33·73	48·57	34·58	41·06	38·4	44·78	42·97	45·58	<0·001	23·73	28·37	28·71	30·26	31·15	36·07	35·05	40·26	<0·001
Sweets	18·41	28·86	18·62	25·85	21·46	30·38	23·64	34·48	0·001	9·32	13·20	13·45	19·15	14·34	20·13	21·1	33·92	<0·001
Sugar-sweetened beverage	59·36	124·54	58·54	97·24	62·42	121·69	62·99	104·52	0·047	28·68	77·36	32·77	64·06	38·93	87·68	49·79	120·7	0·004
Raw nuts and seeds	1	1·12	3·94	3·25	9·85	6·66	24·02	17·78	<0·001	0·93	1·1	4·48	3·6	10·89	7·8	29·59	22·02	<0·001
Roasted and salty nuts and seeds	0·15	0·53	3·44	3·25	8·01	6·84	22·18	23·7	<0·001	0·1	0·46	3·29	3·78	8·02	8·55	24·01	23·85	<0·001

PCAD, premature coronary artery disease.

*Derived from ANOVA.

Multiple-adjusted OR and 95 % CI for PCAD across the quartiles of nuts are shown in Table 3. In the crude model, the highest quartile of nut intake was markedly associated with a decreased risk of PCAD in comparison with the first quartile (OR = 0·26, 95 % CI (0·21, 0·32); *P*_for trend_ < 0·001). The results were almost identical in the adjusted models for demographic and lifestyle variables. Further adjustment for energy and carbohydrate intake did not considerably change the results, while subjects in the fourth quartile had a 71 % lower risk for PCAD compared to those in the first quartile (OR = 0·29, 95 % CI (0·22, 0·37); *P*_for trend_ < 0·001). In the fully adjusted model, adjustment for mediators (HTN, DM, BMI, hypercholesterolemia and aspirin use) only slightly weakened associations but did not affect their significance (OR = 0·32, 95 % CI (0·24, 0·43); *P*_for trend_ < 0·001).

**Table 3:** OR (95 % CI) of premature coronary artery disease risk in different ethnicities according to quartile of nuts and seeds consumption

	Nuts							*P*_for trend_
	Q1 (<4 g/d)		Q2 (4–11·8 g/d)		Q3 (11·8–26·3 g/d)		Q4 (>=26·34 g/d)	
	OR	95 % CI	OR	95 % CI	OR	95 % CI		
Crude model	1	0·91, 1·42	1·14	0·57, 0·87	0·71	0·21, 0·32	0·26	<0·001
Model 1*	1	0·92, 1·5	1·18	0·55, 0·88	0·69	0·21, 0·33	0·26	<0·001
Model 2†	1	0·93, 1·53	1·19	0·55, 0·89	0·7	0·21, 0·33	0·26	<0·001
Model 3‡	1	0·93, 1·53	1·2	0·57, 0·92	0·72	0·22, 0·37	0·29	<0·001
Model 4§	1	0·86, 1·52	1·14	0·6, 1·04	0·79	0·24, 0·43	0·32	<0·001

SSB, sugar-sweetened beverage.

* Model 1: Adjusted for age and sex.

† Model 2: Additionally adjusted for education, smoking (never/ex-smoker/current smoker) and physical activity (METS/min/week).

‡ Model 3: Further adjustment was made for energy (kcal/d) and carbohydrate (% of energy).

§ Model 4: Adjusted for model 3 + BMI (kg/m^2^), hypertension (yes/no), diabetes mellitus (yes/no), hypercholesterolemia (yes/no) and aspirin use (yes/no).

**Fig. 1:**
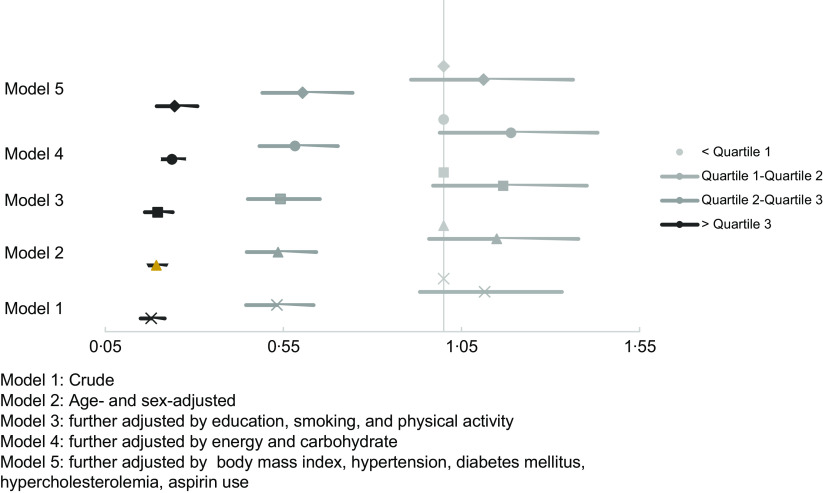
The associations of nut intake with PCAD severity. PCAD severity is the response variable, and the OR and 95 % CI indicate how much closer each quartile of nuts increase into the next category of the PCAD severity. PCAD, premature coronary artery disease

Figure 1 highlights the OR and corresponding 95 % CI for PCAD severity across the quartiles of nut intake. In the crude model, participants in the highest quartile of nut intake had an 82 % lower risk of severe PCAD than those in the first quartile (OR = 0·18, 95 % CI (0·15, 0·22); *P*_for trend_< 0·001). Likewise, the severity of PCAD decreased by 81 % from the reference quartile to the highest quartile after adjusting for age and sex. Higher nut consumption was linked to a lower chance of severe PCAD by 80 % and 76 % in the third and fourth models, respectively, which were adjusted for education, smoking, physical activity, energy and carbohydrate intake. Finally, after full adjustment for confounding variables (including, BMI, HTN, DM, hypercholesterolemia and aspirin use) in Model 5, the highest intake of nut diminished the probability of having severe PCAD by 75 % (OR = 0·25, 95 % CI (0·19, 0·31); *P*_for trend_< 0·001).

## Discussion

In the present case–control study, conducted on different ethnicities of Iranian adults, we found an inverse relationship between nut intake and PCAD. Individuals with higher nut consumption had a 74 % lower risk of PCAD than those in the lower quartile. This association was independent of confounders and mediators. Additionally, an inverse association was observed between nut intake and the severity of PCAD.

Based on previous narrative and systematic reviews, the most frequent modifiable risk factors for PCAD are DM, dyslipidemia, smoking and hypertension^(14,19,20)^. In earlier studies, the significance of prevention in the early development of CVD was emphasised^(21,22)^. The cumulative impact of many risk factors that patients have from early life is substantially related with the occurrence of CVD^(23)^. In a case–control study that assessed the impact of various risk factors on the progression of MI in young adults, dietary habits were the most significant factor^(22)^. On the other hand, unhealthy dietary habits, such as high consumption of red and processed meat, refined grains, and low fruit and vegetable consumption, are more frequent in young and healthy adults with a positive family history of PCAD in comparison with healthy adults without any family history of CAD^(3)^. The development of unhealthy habits during childhood may continue into adulthood, leading to increased cardiovascular risks that previously existed in the family^(3)^. These results imply that risk factors modification, particularly dietary behaviour, can decrease the progression of PCAD.

Our findings corroborate earlier research on the impact of nuts on CVD^(24–28)^. The beneficial impacts of nuts on CVD and its risk factors have been well established^(6,8,10,29–31)^. In a cohort study, higher nut consumption was associated with a 24 % lower risk of cardiovascular mortality and stroke after a follow-up duration of 3·5 years^(25)^. Nut consumption significantly reduced CVD mortality in men by 63 % in a cohort study that specifically investigated the eastern population^(32)^. During a 26-year follow-up, Liu *et al.* evaluated the frequency of nut consumption and the risk of developing CVD, coronary heart disease (CHD) and stroke. They showed that nut consumption of more than 0·5 mg/d decreased the risk of CVD and CHD by 8 and 6 %, respectively. In contrast to participants who did not reduce their daily intake, cutting back on nuts was linked to an increased risk of developing CVD, CHD and stroke^(26)^. Consistently, a systematic review and meta-analysis of twenty prospective cohort studies showed an inverse relationship between nut intake and the incidence and mortality of CVD, CHD and sudden cardiac death. Additionally, participants who consumed nuts had a significantly lower overall mortality rate (Relative Risk (RR): 081; 95 % CI (0·77, 0·85))^(27)^. Moreover, the preventive effects of nuts on CVD risk factors have been investigated, and consistent with earlier studies, a robust favourable impact on hyperlipidemia, HTN^(8,33)^ and metabolic syndrome^(34)^ has been identified. Although prior assessments did not explicitly link nut consumption to CAD severity in young or old individuals, adherence to the Mediterranean diet has been shown to be associated with a lower syntax score and, subsequently, a less severe form of CAD^(35)^. There is still disagreement regarding the precise mechanisms involved in the cardioprotective properties of nuts. The most frequently mentioned reason for this phenomenon is that these foods are high in unique combinations of protein, fibre, minerals, trace elements, MUFA (oleic acid) and PUFA (linoleic acid). Moreover, they contain less than 5 per cent saturated fatty acids (SFA)^(25,26)^. Replacing SFA with MUFA and PUFA helps reduce CVD and mortality^(28)^. It is equally necessary to take into account the beneficial effects of nuts on CVD risk factors, since earlier studies^(36,37)^ have suggested that preventative influence of nuts on CVD and CHD likely stems from them^(36)^. For instance, a review of clinical trials found that nut consumption significantly reduced cholesterol concertation by 10·9 and TAG levels in patients with hypertriglyceridemia^(38)^. In addition, the beneficial effects of nuts on other lipoproteins and apo B/apo A ratio levels have also been reported in epidemiological studies^(39–41)^. Higher consumption of different nuts can also decrease the prevalence of hypertension among Koreans^(42)^, Iranians^(33)^ and individuals with metabolic syndrome^(43)^. Another intriguing fact about nuts is that despite their high calorie content, they do not promote obesity or weight gain^(44)^ and, in contrast, can play a role in weight control by boosting satiety^(45)^. Moreover, the high fibre and protein contents of nuts promote thermogenesis, leading to weight loss^(46)^. Nuts also have anti-inflammatory qualities and promote endothelial integrity, thereby attenuating the risk of CVD and CHD^(47)^. The beneficial effects of nuts on gut flora and circulatory metabolism are noteworthy^(10)^. The digestion of nuts, especially walnuts, increases α and β diversity and has a favourable impact on the gut microbiota of participants^(48)^.

Our study is one of the largest investigations to assess CAD in young adults in the Middle East, specifically focusing on individuals who were at a higher risk for early onset CAD. With the inclusion of participants representing ten different ethnicities in Iran, our study offers valuable insights into the characteristics of CAD within this population subgroup. Importantly, our findings have implications beyond the specific demographics of our study population. The comprehensive nature of our study, which encompassed data from fifteen cities across Iran, contributes to the generalisability of our results to other regions with similar risk profiles. By examining a diverse range of ethnicities within Iran, we aimed to capture the potential interplay between genetic factors and CAD susceptibility among individuals at a higher risk for PCAD. The participants’ coronary disease was determined via angiography which is the gold standard for this matter. Additionally, validated questionnaires were used to assess socio-economic status, pre-existing medical conditions and dietary habits. Our study has several limitations. We assessed dietary intake using the FFQ which causes inevitable measurement biases. It is impossible to link the positive results of nuts in our study to a specific kind of nuts because we did not assess various nuts. Furthermore, due to the small sample size in several ethnic categories, we were unable to quantify the PCAD risk across them. In our study, we did not use energy-adjusted analysis using residuals. Instead, we accounted for energy intake as a covariate in our regression models, alongside other relevant variables. We understand that conducting energy-adjusted analysis using residuals could have been an additional sensitivity analysis to test the reliability of our results. However, considering the complexity of our dataset and the extensive range of covariates already incorporated in our models, we believe that our chosen approach adequately addresses any potential influence from energy intake. Additionally, it is worth mentioning that previous studies indicated comparable outcomes between the two methods, suggesting that they are equally effective in achieving the desired outcome^(49)^. Another drawback of case–control studies is the increased propensity of the case group to remember the exposure (in this study, eating nuts) compared with the control group without the outcome. This could lead to erroneous drawing, including conclusions that claim an inverse relationship exist between exposure and disease. Finally, because the survey was a case–control in nature, it was not feasible to establish a causal link.

### Conclusion

Our study suggests a cardioprotective role of nuts in reducing PCAD risk among Iranian adults. This association is strong and independent of covariates and mediators. Further large-scale clinical trials are needed to clarify the precise mechanisms underlying this association.
